# The Ohio State Journal of Dental Science

**Published:** 1882-02

**Authors:** 


### THE OHIO STATE JOURNAL OF DENTAL SCIENCE.

This publication, which for the first year was a bi-monthly; notwithstanding it started at a high point of excellence, it not only sustained its position, but came out at the end of the year on the ascending grade; and then so easy did the work seem to be that the editor and publishers decided to make it a monthly, and the first number of the second volume is equal to, if not better, than any of its predecessors. The JOURNAL is a lusty and healthy yearling, and will doubtless make its way through the world, and if anybody does not want to be run over, or run through, he had better keep out of the way. George strikes right out from the shoulder, and he knows where to hit to make it tell. He has studied anatomy. *Long live the* JOURNAL.

